# Nutritional Risk, Depression, and Physical Function in Older People Living Alone

**DOI:** 10.3390/healthcare12020164

**Published:** 2024-01-10

**Authors:** Jeong-Hye Park, Se-Won Kang

**Affiliations:** 1 Department of Nursing, Gyeongsang National University, 33 Dongjin-ro, Jinju-si 52725, Republic of Korea; masternur@gnu.ac.kr; 2 Department of Nursing, Dongseo University, 47 Jurye-ro, Sasang-gu, Busan 47011, Republic of Korea

**Keywords:** nutrition, depression, physical function, older adults

## Abstract

This study investigated depression and physical function as factors associated with nutritional risk in older adults living alone. The study included 2896 people 65 years or older who lived alone. Data were collected in South Korea between September and November 2020. Descriptive statistics, a chi-squared test, independent samples *t*-test, weighted multiple regression analysis, and binary logistic regression analysis were performed using IBM SPSS for Windows ver. 23.0. In this study, 44.8% of participants were in the nutritional risk group. Furthermore, 60.9% of those at risk for depression, 75.1% of those with instrumental activities of daily living (IADLs) dependency, and 59.1% of those with chewing limitations were at nutritional risk. The factors that increased nutritional risk in the weighted multiple regression analysis were depression (β = 0.27, *p* < 0.001), chewing limitations (β = 0.12, *p* < 0.001), IADL dependency (β = 0.09, *p* < 0.001), and basic physical movement (β = 0.04, *p* = 0.020). Binary logistic regression analysis showed that those with IADL dependency had a 2.59 times higher nutritional risk than those with IADL non-dependency (*p* < 0.001). The nutritional risk group had a higher risk of depression (2.01 times higher [*p* < 0.001]), chewing limitations (1.76 times higher [*p* < 0.001]), and basic physical movement limitations (1.35 times higher [*p* = 0.009]) than the good nutritional group. Therefore, nutritional screening is required of older individuals living alone. To mitigate nutritional risks, it is necessary to assess depression and physical function, including IADL dependency.

## 1. Introduction

The world’s population is aging rapidly owing to medical advancements and increased life expectancy. In particular, Korea’s elderly population is growing rapidly. In 2022, individuals aged 65 years or older accounted for 17.4% of the population; this figure is expected to increase to 29.9% by 2035 [1]. In Korean society, the number of older adults living alone is increasing annually because of social, economic, and cultural changes such as a weakening sense of support for parents, an increase in women’s economic activities, and nuclear families. The proportion of households with individuals aged 65 years or older living alone increased gradually from 6.4% in 2015 to 9.1% in 2022 and is expected to continue to increase in the future owing to changes in family type and lifestyle [2]. Older adults living alone are vulnerable to economic conditions and poor physical, mental, and social health. Moreover, they tend to have lower levels of education and economic status than those living with their spouse or children [3]. The prevalence of chronic diseases, multiple chronic diseases, depressive symptoms, and cognitive decline is relatively high, as is the rate of unmet medical care needs, raising concern about worsening physical and mental health problems in older adults living alone [4]. Additionally, accident, fall, and abuse rates are higher among older adults living alone than among those with other living arrangements [5]. As such, older people living alone are vulnerable to physical, psychological, and physiological vulnerability due to aging; therefore, multifaceted social attention and support is needed to maintain their daily function and optimize their health [6].

Optimizing one’s nutritional status is important because poor nutritional status in old age leads to problems such as falls, infection, disease, impaired wound healing, long-term care facility admission, prolonged hospitalization, and disease-related morbidity and mortality [7]. However, most older adults experience poor health conditions such as physiological aging, poor teeth, loss of vision, decreased saliva secretion and gastrointestinal absorption, a decreased support system that reduces social interactions, decreased appetite due to depression, chronic diseases, and low economic status. Most nutrients are insufficiently consumed [8]. According to a survey in Korea, 27.7% of the older population requires nutritional management and 8.8% are at high risk of at least one nutritional deficiency requiring immediate improvement [9]. Older adults living alone are often economically disadvantaged and unable to purchase high-quality food ingredients. The risk of malnutrition is especially high when meal preparation becomes difficult, and meals are frequently skipped because of a lack of motivation to eat [10]. Checking for such factors is necessary to help older individuals living alone to maintain an adequate nutritional status.

Nutritional problems in older adults are also related to psychological problems such as depression and loneliness. Depression decreases an individual’s participation in social activities [11]. Depression in older individuals manifests as symptoms of memory decline or loss of appetite rather than emotional aspects such as sadness and is often mistaken for dementia or other diseases [12]. This suggests that depression in older people must be assessed and treated differently from depression in younger individuals. Moreover, unbalanced nutritional intake owing to depression can also cause nutritional deficiencies and health problems in older adults.

Limitations or dependency on others for one’s daily activities and functioning may be major factors contributing to nutritional risk in older populations. In particular, an unhealthy oral status and lack of independence affect an individual’s meal preparation, food intake, and eating habits [13]. Maintaining meal quantity and quality may be difficult due to chewing difficulties, especially in old age [14]. The foods consumed by older people vary according to the condition of their mouth, teeth, or gums; older people who have difficulty chewing tend to choose foods that are easy to chew, and their nutrient intake may become unbalanced because of a reduced intake of fruits and vegetables [15]. Thus, chewing ability in older individuals affects their nutritional intake, digestion, and absorption of nutrients, resulting in malnutrition and unbalanced eating [16].

The nutritional status of older adults is influenced by various factors, such as age, education level, economic status, excessive drinking, smoking, exercise, body mass index (BMI), chronic diseases, polypharmacy, poor vision, memory complaints, depression, activities of daily living (ADLs), instrumental ADLs (IADLs), and accessibility to community facilities [14,17]. However, most studies to date focused on older community-dwelling people, while research is lacking on the factors related to nutritional deficiencies in older people in single-person households. Therefore, large-scale studies are needed to increase the effectiveness of nutritional deficiency interventions for older adults living in single-person households. This study aimed to assess the nutritional status of older adults living alone in the community and identify factors affecting nutritional risk, particularly depression and physical function.

### Study Purpose

This study aimed to identify the nutritional risk and related factors in older adults living alone in the community by focusing on depression and physical function. Its specific objectives were as follows:

1. Investigate differences in nutritional risk according to participant characteristics, depression, and physical function;
2. Identify depression and physical function factors related to nutritional risk.

## 2. Materials and Methods

### 2.1. Study Design

This secondary data analysis investigated the factors related to nutritional risk among older adults living alone.

### 2.2. Study Participants

The data of 2896 study participants were selected according to the following inclusion criteria: (1) ≥65 years of age; (2) living alone; (3) ability to respond independently to the survey (i.e., no cognitive impairment); and (4) availability of all data for key variables.

### 2.3. Data Collection

Raw data were obtained from the 2020 Survey on the Status of the Elderly, which was provided and used according to data use procedures presented by the Health and Welfare Data Portal [18]. The Senior Citizen Survey is conducted every 3 years for older individuals in Korea to investigate their living conditions and welfare needs and prepare basic data for establishing welfare policies for older people.

The numbers of survey districts and households were determined using stratified cluster sampling. A two-stage cluster sampling method was employed to prevent undersampling in areas with smaller populations, thereby ensuring a representative sample. Raw data were sampled by stratifying the entire country into 17 cities and provinces and then stratifying the nine provincial regions, excluding eight particular metropolitan cities, by dividing them into the units of a local administrative districts belonging to cities or towns. The data were collected through one-on-one interviews conducted by trained investigators between September and November 2020.

### 2.4. Study Variables

#### 2.4.1. Nutritional Risk

Nutritional risk was assessed using the Korean version of the Nutrition Screening Initiative DETERMINE Checklist (NSI) [19]. This tool consists of 10 questions that are used to initially assess the nutritional health status of older adults and screen for malnutrition risk. The total score is calculated by assigning the number of “yes” responses to each question and a set weight (1–4 points for each question) to the “yes” questions. The total score is 21 points; the higher the score, the greater the nutritional risk. Depending on the total score, the level of nutritional risk is classified as “good” in cases of ≤2 points (Good group) and “at risk” in cases of ≥3 points (Risk group). In this study, the reliability of the tool had a Cronbach’s alpha of 0.81.

#### 2.4.2. Depression

Depression was measured using the Korean version of the short form of the Geriatric Depression scale [20]. This tool is the Korean version of the Geriatric Depression Scale, which is used worldwide to screen older populations for depression. The scale consists of 15 questions and is calculated by adding the scores of yes answers (1 point) with binary options of “yes” and “no.” The score is based on 15 points in total; if the total score is ≥5, the individual is considered at risk of depression [8,21]. The tool’s reliability had a Cronbach’s alpha of 0.85.

#### 2.4.3. Physical Function

Physical function variables were derived from previous studies [22,23,24,25]. Physical function refers to one’s ability to perform physical movements or actions ranging from simple physical movements (e.g., walking across a room) to more complex activities (e.g., ADLs) required for independent living. This differentiates basic movements from complex activities. Functional status includes questions related to one’s ability to perform activities such as self-reporting, proxy report (e.g., spouse, children), direct observation, and direct measurement [22]. Accordingly, the variables of physical function in this study include chewing related to eating, repeated chair stands, and basic physical movements as simple physical movements as well as ADLs and IADLs as complex activities. Chewing, basic physical movements, ADLs, and IADLs were measured through self-reporting, while repeated chair stands were assessed through direct measurements.

(1) Chewing

One question asked whether the participants experienced any discomfort in daily life when chewing meat or hard objects (using dentures if needed). Possible answers included “good” or “limited”.

(2) Repeated chair stands

Repeated chair stands involved standing up and sitting down on a chair five times without using the hands [25]. This was measured as “Good–Completed performance” or “Limited–Could not complete five times, unable to attempt due to bedridden status or other disabilities”.

(3) Basic physical movement

Basic physical movements require muscle strength and range of motion [22,23,24]. This area consists of five items, each rated on a four-point Likert scale ranging from 0 (not at all difficult) to 3 (very difficult). Higher scores indicate greater difficulty with a physical activity. The five items were walking 400 m, going up 10 stairs without stopping, bending, squatting, or bending the knees, reaching a place higher than your head, and moving or lifting an object weighing about 8 kg (such as an armchair). In this study, the tool’s reliability had a Cronbach’s alpha of 0.88.

(4) ADLs and IADLs

ADLs and IADLs were assessed using the following question: “During the past week, to what extent did you need help from others to perform (XXX activity)?” This was rated as not at all needed, partially needed, and completely needed. The ADLs section consisted of 7 items, while the IADLs section consisted of 10 items; when all items were rated as not at all needed, the participant was considered independent, whereas when even one item was rated as partially needed or completely needed, the participant was considered dependent. The reliability of the ADLs and IADLs in this study had Cronbach’s alpha values of 0.94 and 0.95, respectively.

The seven items of the Korean (K)-ADLs [26] include selecting and donning appropriate clothes; maintaining one’s face/hair/dental hygiene; bathing and showering oneself; feeding oneself; getting out of bed and leaving the bedroom independently; getting to and from and using the toilet appropriately; and controlling one’s urine and bowel movements. The 10 items of the Korean (K)-IADLs [27] include grooming oneself, house cleaning and tidying, preparing meals, doing laundry, shopping for items required for daily life (clothing, groceries, etc.), making and receiving phone calls, obtaining and taking medications as directed, paying and managing one’s financial assets, leaving home for short distances, and using transportation.

#### 2.4.4. Adjusted Variables

The adjusted variables included general characteristics, smoking and drinking habits, and sensory factors. General characteristics included sex, age, education level, homeownership, annual income, number of chronic diseases, number of medications taken to treat chronic diseases, and BMI (kg/m^2^). In this study, chronic disease refers to a disease that has been present for more than 3 months after being diagnosed by a doctor, such as hypertension, diabetes mellitus, and heart disease. A BMI of <18.5 was classified as underweight, 18.5–22.9 as normal, 23–24.9 as pre-obesity, and ≥25 as obese according to the BMI criteria of the 2022 Korean Obesity Society Obesity Treatment Guidelines [28]. Smoking was measured using the question “Do you currently smoke?” Drinking was measured using a six-point Likert scale (0: none; 1: less than once a month; 2: once a month; 3: twice a month; 4: once a week; and 5: more than twice a week). The question was, “How often have you had a drink in the past year?” Sensory factors included vision and hearing. Each question was asked with respect to their vision and hearing and whether these sensory functions cause any discomfort in daily life (after the application of braces, glasses, lenses, magnifying glasses, and hearing aids). The responses were measured as good or limited. An example of vision related to daily life was “watching TV or reading newspapers”. An example of hearing was “talking on the phone or talking to the person next to you”.

### 2.5. Ethical Considerations

The study was approved by the Ethics Committee of the Korea Institute for Health and Social Affairs (no. 2020–36) and conducted by trained investigators after written informed consent was provided by each participant. The data were easily obtained via the Korea Institute for Health and Social Affairs Health and Welfare Data Portal. Identifying the participants was impossible, as no personal information about the respondents was available.

### 2.6. Data Analysis

The data were analyzed using SPSS Statistics (version 23.0; IBM, Armonk, NY, USA).

(1) Participant characteristics, depression, and physical function are presented as frequencies, percentages, and means and standard deviations. Differences in nutritional risk among variables were analyzed using the chi-squared test and independent sample *t*-tests with consideration of normality.
(2) Weighted multiple regression and binary logistic regression analyses were used to identify factors related to nutritional risk.
(3) The statistical significance level of this study was *p* < 0.05.

## 3. Results

### 3.1. Differences in Nutritional Risk by Participant Characteristics, Depression, and Physical Function

The differences in nutritional risk according to participant characteristics, depression, and physical function are presented in Table 1 and Table 2.

Of the 2896 participants, 55.2% (n = 1600) were in the good nutritional group and 44.8% (n = 1296) were in the nutritional risk group. A total of 44.1% of the men and 45.2% of the women belonged to the nutritional risk group. The average number of diagnosed chronic diseases was 2.1 ± 1.53, while the mean number of medicines taken was 2.0 ± 1.59. In terms of participant characteristics, variables that showed statistically significant differences in nutritional risk were age (t = −7.40, *p* < 0.001), educational level (χ^2^ = 37.10, *p* < 0.001), homeownership (χ^2^ = 12.24, *p* < 0.001), annual income (t = 5.01, *p* < 0.001), number of chronic diseases (t = −13.72, *p* < 0.001), number of medicines taken (t = −13.24, *p* < 0.001), and BMI (χ^2^ = 8.55, *p* = 0.036). Overall, 65.7% (n = 1901) of the participants had a BMI above the normal range, while 45.9% (n = 873) of those were at nutritional risk. Regarding depression and physical function (Table 2), all variables differed significantly between study groups. In particular, significant differences were seen in depression (χ^2^ = 191.15, *p* < 0.001), chewing (χ^2^ = 185.62, *p* < 0.001), and IADLs (χ^2^ = 145.64, *p* < 0.001). Furthermore, 60.9% of those at risk for depression, 75.1% of those with IADL dependency, and 59.1% of those with chewing limitations were at nutritional risk. 

### 3.2. Factors Related to Nutritional Risk by Depression and Physical Function Status

Weighted multiple regression and binary logistic regression analyses were performed to identify the factors related to nutritional risk. The regression model had a tolerance limit of 0.56–0.83, which was >0.1, and the variance inflation factor (VIF) was 1.21–1.79, which was below the standard value of 10, so there was no problem with multicollinearity between the independent variables. The results of the weighted multiple regression analysis are presented in Table 3, while those of the binary logistic regression analysis are shown in Table 4.

Factors that significantly increased nutritional risk were depression (β = 0.27, *p* < 0.001), chewing limitations (β = 0.12, *p* < 0.001), IADL dependency (β = 0.09, *p* < 0.001), and basic physical movement limitations (β = 0.04, *p* = 0.020) (Table 3). Compared with the good nutritional group, the nutrition risk was 2.59 times higher in participants with IADL dependency (*p* < 0.001, 95% confidence interval [CI]: 1.847–3.630), 2.01 times higher in depressed participants (*p* < 0.001, 95% CI: 1.699–2.389), 1.76 times higher in those with chewing limitations (*p* < 0.001, 95% CI: 1.435–2.146), and 1.35 times higher in those with basic physical movement limitations (*p* = 0.009, 95% CI: 1.078–1.701) (Table 4).

## 4. Discussion

### 4.1. Nutritional Risk, Depression, and Physical Function of Participants

This study revealed the factors contributing to high nutritional risk related to depression and physical function among community-dwelling people aged 65 years or older living alone. Among the participants, 44.8% were at nutritional risk. This result is lower than the nutritional risk group of 78.9% reported by a study of older Korean men living alone [9] but higher than the 30.9% reported by a study of community-dwelling older people in Singapore [29]. The nutritional risk of 44.8% in the older population of single-person households in the community is fairly high; therefore, community intervention is necessary.

Additionally, 38.5% of the participants were at risk of depression. This rate was higher than that of depression (26.9%) among older adults living alone in Shanghai, China [30]. Depression in older people living alone is also linked to social problems such as dying alone; therefore, social support systems must be carefully examined. The examination of physical functions revealed that chewing limitations, repeated chair stands, and basic physical movement limitations affected 30.9–81.4% of participants, including the ADLs of 4.7% and the IADLs of 11.9%. The average participant age was 75 years; most were entering the later stages of geriatric life. The most noticeable aspect of the aging process is the decline in physical function; the participants in this study showed partially limited physical function. The difference in the distribution of depression and physical function was that the nutritionally good group had no risk of depression compared to the high-risk group and maintained good physical functional status. This finding is similar to a study conducted of 400 older adults in Bangladesh [31], which reported that the relative risk of exposure to depression was high when nutritional status was cautious or dangerous. In addition, the results were similar to those of Byeon’s study, which showed that poor a nutritional status was related to frailty and decreased physical function [32].

As the number of single-person households continues to increase, health problems are expected to increase owing to nutritional vulnerability. A comprehensive multidisciplinary approach should be prioritized in terms of assessing social interests and efforts in nutrition, nursing, health, and welfare.

### 4.2. Factors Associated with Participants’ Nutritional Risk

In the multiple regression analysis, depression, chewing limitations, basic physical movement limitations, and IADL dependency were factors that increased nutritional risk. Higher levels of depression, chewing limitations, basic physical movement limitations, greater IADL dependency, and higher nutritional risk were observed. The logistic regression analysis revealed that the participants’ nutritional risk was 2.01 times higher if depressed, 1.76 times higher in the presence of chewing limitations, 1.35 times higher in the presence of basic physical movement limitations, and 2.59 times higher in cases of IADL dependency.

Depression is also a major risk factor for poor nutrition. This is similar to the results of an investigation of the relationship between malnutrition and depression among 4916 people over 60 years of age living in a Chinese community [33] in that depression was 1.311 times higher in people with versus without malnutrition. A study of 600 older adults in Bangladesh [8] showed that malnourished older adults were 3.155 times more likely to be depressed than well-nourished older adults, similarly to our results. Kilmova et al. [34] conducted a meta-analysis of six studies examining the dietary patterns and nutritional intake of older adults and reported that they were associated with depressive symptoms. In this study, we simply analyzed nutritional risk status; however, it is also necessary to analyze the dietary patterns and degree of skipping meals among older people living alone to determine their relationship with depression.

Chewing limitations are also a major nutritional risk factor. This finding is similar to a study that tracked dental vulnerability and nutritional risk in 466 older Japanese people [35] and found that oral frailty worsened nutritional status by 2.24 times. Additionally, in a survey of 715 people aged 65–91 years in Otassha, Japan [36], oral hypofunction increased malnutrition threefold. The degree of masticatory ability according to the condition of an older person’s teeth or the number of natural teeth plays an important role in maintaining an appropriate nutritional intake and is believed to be a major factor in our study results.

Basic physical movement limitations are also a major factor in nutritional risk. This was the result of a study targeting 222 people aged 65 years or older in the rural Peruvian Andes area [37]. Malnutrition was associated with a 4.94-times-poorer performance in the short physical performance battery and 2.73-times-poorer performance in the 6 min walking test. A study of 286 older adults by Ramsey et al. [38] reported that malnutrition is strongly related to gait speed. Although the measurement items are somewhat different, the basic physical movement investigated in this study included walking 400 m, climbing 10 stairs without stopping, bending, squatting or bending the knees, reaching a place higher than your head, and lifting. As shown in performance limitations including move an object weighing about 8 kg, malnutrition is believed to affect dynamic physical performance.

IADL dependency was the main nutritional risk factor. This finding is similar to that of a study by Nagai et al. [39] of 468 community-dwelling older individuals in which a decline in IADLs was associated with a relative nutritional risk of 2.22. By tracking 2075 patients 60 years or older in Singapore for 4–5 years [40], nutritional deterioration was the equivalent of a 3.22-fold increase in IADL/ADL disability. Nutritional risk affects health and has adverse functional outcomes.

The results of this study suggest that not only physical function but also mental status should also be considered as a factor of nutritional risk in nutritional risk assessments of older people. Chewing and basic physical movements were important factors for nutritional risk; however, depression and IADLs were even more meaningful. Therefore, it is essential to assess these factors to prevent nutritional risk.

The nutritional status of the elderly affects both functional status and quality of life. The risk rates and predictive factors for nutritional disorders shown in this study indicate the need for social attention and a comprehensive approach to improving nutritional status. Therefore, aspects of mental and physical functioning should be considered in the development of healthcare interventions to maintain and improve the nutritional status of older adults living alone.

### 4.3. Study Limitations

This cross-sectional study had several limitations. First, it used secondary data and had limited ability to set the variables related to nutritional risk, depression, and physical function in older adults. Second, because this was a cross-sectional study, caution is needed when interpreting causal relationships. Future studies should address these limitations using longitudinal data to identify the causal relationships between predictive factors and nutritional risk. Third, this study included only older people living alone; however, the characteristics of the variables can be revealed more clearly in future studies of older people who do or do not live alone. Fourth, although this study measured nutritional status using a tool validated by trained investigators, the validity of nutritional status assessments may be problematic because some items were assessed through self-reporting. Therefore, in future research, it would be beneficial to add objective indicators (e.g., biochemical indicators) that can be used to evaluate an individual’s physical condition in addition to self-reported nutritional evaluation scales.

## 5. Conclusions

This study shows the nutritional risk of older people living alone in the community and the factors related to depression and physical function. One of these two could put older people at nutritional risk. Importantly, nutritional risk was more strongly associated with depression and IADL dependency than with chewing and basic physical activity limitations. Therefore, screening the nutritional status of older individuals in single-person households is essential. Ensuring this demographic has no problems with depression or independent daily life, especially IADLs, is necessary. These issues should be considered when establishing welfare policies for the increasing number of older single-person households.

## Figures and Tables

**Table 1:** Differences in nutritional risk according to participant characteristics.

Variable		Nutritional Risk			χ^2^ or t(*p*)
		Total (n = 2896)	Good Group (n = 1600)	Risk Group (n = 1296)	
		N (%) or Mean ± SD			
Sex	Men	1151 (39.7)	643 (55.9)	508 (44.1)	0.29 (0.593)
	Women	1745 (60.3)	957 (54.8)	788 (45.2)	
Age (years)		75.3 ± 6.80	74.5 ± 6.67	76.3 ± 6.83	−7.40 (<0.001)
Educational level	≤Primary education	1751 (60.5)	889 (50.8)	862 (49.2)	37.10 (<0.001)
	Secondary education	1084 (37.4)	669 (61.7)	415 (38.3)	
	Higher education	61 (2.1)	42 (68.9)	19 (31.1)	
Homeownership	Homeowner	1944 (67.1)	1118 (57.5)	826 (42.5)	12.24 (<0.001)
	Other	952 (32.9)	482 (50.6)	470 (49.4)	
Annual income *		1388.4 ± 2365.60	1571.4 ± 2986.18	1162.5 ± 1186.18	5.01 (<0.001)
Chronic disease (number)		2.1 ± 1.53	1.7 ± 1.23	2.5 ± 1.73	−13.72 (<0.001)
Taking medicines (number)		2.0 ± 1.59	1.7 ± 1.25	2.5 ± 1.84	−13.24 (<0.001)
Body mass index (kg/m^2^)	<18.5	76 (2.6)	34 (44.7)	42 (55.3)	8.55 (0.036)
	18.5–22.9	919 (31.7)	538 (58.5)	381 (41.5)	
	23–24.9	1172 (40.5)	631 (53.8)	541 (46.2)	
	≥25	729 (25.2)	397 (54.5)	332 (45.5)	
Smoking	No	2681 (92.6)	1493 (55.7)	1188 (44.3)	2.82 (0.093)
	Yes	215 (7.4)	107 (49.8)	108 (50.2)	
Drinking	No	2021 (69.8)	1096 (54.2)	925 (45.8)	2.80 (0.094)
	Yes	875 (30.2)	504 (57.6)	371 (42.4)	
Vision	Good	1820 (62.8)	1120 (61.5)	700 (38.5)	78.38 (<0.001)
	Limited	1076 (37.2)	480 (44.6)	596 (55.4)	
Hearing	Good	2090 (72.2)	1253 (60.0)	837 (40.0)	67.19 (<0.001)
	Limited	806 (27.8)	347 (43.1)	459 (56.9)	

* Korean won, tens of thousands; SD, standard deviation.

**Table 2:** Differences in nutritional risk according to depression and physical function status.

Variable		Nutritional Risk			χ^2^ or t(*p*)
		Total (n = 2896)	Good Group (n = 1600)	Risk Group (n = 1296)	
		N (%) or Mean ± SD			
Depression	No risk	1781 (61.5)	1164 (65.4)	617 (34.6)	191.15 (<0.001)
	Risk	1115 (38.5)	436 (39.1)	679 (60.9)	
Physical function					
Chewing	Good	1633 (56.4)	1083 (66.3)	550 (33.7)	185.62 (<0.001)
	Limited	1263 (43.6)	517 (40.9)	746 (59.1)	
Repeated chair stands	Good	2000 (69.1)	1209 (60.5)	791 (39.6)	70.73 (<0.001)
	Limited	896 (30.9)	391 (43.6)	505 (56.4)	
Basic physical movement	Good	539 (18.6)	382 (70.9)	157 (29.1)	65.38 (<0.001)
	Limited	2357 (81.4)	1218 (51.7)	1139 (48.3)	
	Score (mean ± SD)	4.7 ± 3.81	4.0 ± 3.62	5.5 ± 3.87	−10.77 (<0.001)
ADLs	Independent	2761 (95.3)	1570 (56.9)	1191 (43.1)	62.47 (<0.001)
	Dependent	135 (4.7)	30 (22.2)	105 (77.8)	
IADLs	Independent	2551 (88.1)	1514 (59.3)	1037 (40.7)	145.64 (<0.001)
	Dependent	345 (11.9)	86 (24.9)	259 (75.1)	

ADLs, activities of daily living; IADLs, instrumental activities of daily living; SD, standard deviation.

**Table 3:** Multiple regression analysis of nutritional risk by depression and physical function status.

Variable		Nutritional Risk		
		β	SE	*p*
Depression		0.27	0.02	<0.001
Physical function	Chewing	0.12	0.14	<0.001
	Repeated chair stands	−0.04	0.14	0.050
	Basic physical movement	0.04	0.16	0.020
	ADLs	−0.00	0.31	0.858
	IADLs	0.09	0.22	<0.001

R^2^: 0.227; Adjusted R^2^: 0.222; F(*p*): 46.86 (<0.001); Tolerance: 0.56–0.83; Variance inflation factor: 1.21–1.79; ADLs, activities of daily living; IADLs, instrumental activities of daily living. Adjusted variables included age, educational level, home ownership status, annual income, chronic disease, taking medicine, body mass index, vison, and hearing.

**Table 4:** Binary logistic regression of nutritional risk by depression and physical function status.

Variable			Nutritional Risk			
			B	*p*	OR	95% CI
Depression		No risk	Reference			
		Risk	0.70	<0.001	2.01	1.699–2.389
Physical function	Chewing	Good	Reference			
		Limited	0.56	<0.001	1.76	1.435–2.146
	Repeated chair stands	Good	Reference			
		Limited	−0.05	0.623	0.95	0.775–1.165
	Basic physical movement *	Good	Reference			
		Limited	0.30	0.009	1.35	1.078–1.701
	ADLs	Independent	Reference			
		Dependent	−0.16	0.549	0.85	0.499–1.447
	IADLs	Independent	Reference			
		Dependent	0.95	<0.001	2.59	1.847–3.630

χ^2^(*p*) = 460.46(<0.001); Nagelkerke R^2^ = 0.197; Hosmer and Lemeshow χ^2^(*p*) = 9.24(0.323); ADLs, activities of daily living; IADLs, instrumental activities of daily living; OR, odds ratio; CI, confidence interval; * Basic physical movement was defined as Good (group with score of 0) and Limited (group with score of ≥1). Adjusted variables included age, educational level, homeownership, annual income, chronic disease, taking medicine, body mass index, vision, and hearing.

## Data Availability

All data are contained within the article.

## References

[1] Korean Statistical Information Service Population. https://kosis.kr/statHtml/statHtml.do?orgId=101&tblId=DT_1BPA002&checkFlag=N.

[2] Korean Statistical Information Service Proportion of Elderly Households Living Alone. https://kosis.kr/statHtml/statHtml.do?orgId=101&tblId=DT_1YL12701.

[3] Lage I., Braga F., Almendra M., Meneses F., Teixeira L., Araújo O. (2023). Older people living alone: A predictive model of fall risk. Int. J. Environ. Res. Public Health.

[4] Lee H.J. (2021). Relationship between naming abilities, hearing handicap, and depression in community-dwelling elderly. J. Korea Converg. Soc..

[5] Lette M., Stoop A., Nijpels G., Baan C., de Bruin S., van Hout H. (2022). Safety risks among frail older people living at home in the Netherlands—A cross-sectional study in a routine primary care sample. Health Soc. Care Community.

[6] Ishikawa M., Yokoyama T. (2022). The relationship between individual and environmental factors related to health, nutritional status, and diet in elderly people living alone in Japan. Nutr. Rev..

[7] Eckert C., Gell N.M., Wingood M., Schollmeyer J., Tarleton E.K. (2021). Malnutrition risk, rurality, and falls among community-dwelling older adults. J. Nutr. Health Aging.

[8] Islam M.Z., Disu T.R., Farjana S., Rahman M.M. (2021). Malnutrition and other risk factors of geriatric depression: A community-based comparative cross-sectional study in older adults in rural Bangladesh. BMC Geriatr..

[9] Jang H.Y., Kim J.H. (2020). Factors influencing malnutrition in elderly men living alone. Korean Data Anal. Soc..

[10] Norman K., Haß U., Pirlich M. (2021). Malnutrition in older adults-recent advances and remaining challenges. Nutrients.

[11] Kim C., Chang E.J., Kim C.Y. (2021). Regional differences in the effects of social relations on depression among Korean elderly and the moderating effect of living alone. J. Prev. Med. Public Health.

[12] Chen Y. (2022). Risk factors for depression among older adults living alone in Shanghai, China. Psychogeriatrics.

[13] Kotronia E., Wannamethee S.G., Papacosta A.O., Whincup P.H., Lennon L.T., Visser M., Weyant R.J., Harris T.B., Ramsay S.E. (2019). Oral health, disability and physical function: Results from studies of older people in the United Kingdom and United States of America. J. Am. Med. Dir. Assoc..

[14] Woo J., Tong C., Yu R. (2018). Chewing difficulty should be included as a geriatric syndrome. Nutrients.

[15] Kyrdalen I.L., Thingstad P., Sandvik L., Ormstad H. (2019). Associations between gait speed and well-known fall risk factors among community-dwelling older adults. Physiother. Res. Int..

[16] Velázquez-Olmedo L.B., Borges-Yáñez S.A., Andrade Palos P., García-Peña C., Gutiérrez-Robledo L.M., Sánchez-García S. (2021). Oral health condition and development of frailty over a 12-month period in community-dwelling older adults. BMC Oral Health.

[17] Besora-Moreno M., Llauradó E., Tarro L., Solà R. (2020). Social and economic factors and malnutrition or the risk of malnutrition in the elderly: A Systematic review and meta-analysis of observational studies. Nutrients.

[18] MicroData Intergrated Service Elderly Survey. https://mdis.kostat.go.kr/index.do.

[19] Sinnett S., Bengle R., Brown A., Glass A.P., Johnson M.A., Lee J.S. (2010). The validity of nutrition screening initiative DETERMINE Checklist responses in older Georgians. J. Nutr. Elder..

[20] Bae J.N., Cho M.J. (2004). Development of the Korean version of the geriatric depression scale and its short form among elderly psychiatric patients. J. Psychosom. Res..

[21] Fatima M., Sehar A., Ali M., Iqbal A., Shaukat F. (2019). Incidence of depression among community dwelling healthy elderly and the predisposing socio-environmental factors. Cureus.

[22] Painter P., Stewart A.L., Carey S. (1999). Physical functioning: Definitions, measurement, and expectations. Adv. Ren. Replace. Ther..

[23] Béland F., Zunzunegui M.V. (1999). Predictors of functional status in older people living at home. Age Ageing.

[24] Wilkins C.H., Roe C.M., Morris J.C. (2010). A brief clinical tool to assess physical function: The mini-physical performance test. Arch. Gerontol. Geriatr..

[25] Short Physical Performance Battery (SPPB)—Protocol. https://research.ndorms.ox.ac.uk/prove/documents/assessors/outcomeMeasures/SPPB_Protocol.pdf.

[26] Won C.W., Rho Y.G., Kim S.Y., Cho B.R., Lee Y.S. (2002). The validity and reliability of Korean activities of daily living (K-ADL) scale. J. Korean Geriatr. Soc..

[27] Won C.W., Rho Y.G., Sunwoo D., Lee Y.S. (2002). The validity and reliability of Korean instrumental activities of daily living (K-IADL) scale. J. Korean Geriatr. Soc..

[28] Kim K.K., Haam J.H., Kim B.T., Kim E.M., Park J.H., Rhee S.Y., Jeon E., Kang E., Nam G.E., Koo H.Y. (2023). Evaluation and treatment of obesity and its comorbidities: 2022 update of clinical practice guidelines for obesity by the Korean Society for the Study of Obesity. J. Obes. Metab. Syndr..

[29] Wei K., Nyunt M.S.Z., Gao Q., Wee S.L., Ng T.P. (2017). Frailty and malnutrition: Related and distinct syndrome prevalence and association among community-dwelling older adults: Singapore longitudinal ageing studies. J. Am. Med. Dir. Assoc..

[30] Yang R., Wang H., Tracy E.L., Jo Y.J., Sward K.A., Edelman L.S., Demiris G. (2023). What is the relationship between falls, functional limitations, and depressive symptoms among Chinese older adults? The role of living alone. Maturitas.

[31] Alam M.R., Karmokar S., Reza S., Kabir M.R., Ghosh S., Mamun M.A.A. (2021). Geriatric malnutrition and depression: Evidence from elderly home care population in Bangladesh. Prev. Med. Rep..

[32] Byeon H. (2019). Relationship between physical activity level and depression of elderly people living alone. Int. J. Environ. Res. Public Health.

[33] Wei J., Fan L., Zhang Y., Li S., Partridge J., Claytor L., Sulo S. (2018). Association between malnutrition and depression among community-dwelling older Chinese adults. Asia Pac. J. Public Health.

[34] Klimova B., Novotny M., Valis M. (2020). The impact of nutrition and intestinal microbiome on elderly depression-a systematic review. Nutrients.

[35] Iwasaki M., Motokawa K., Watanabe Y., Shirobe M., Inagaki H., Edahiro A., Ohara Y., Hirano H., Shinkai S., Awata S.A. (2020). A two-year longitudinal study of the association between oral frailty and deteriorating nutritional status among community-dwelling older adults. Int. J. Environ. Res. Public Health.

[36] Iwasaki M., Motokawa K., Watanabe Y., Shirobe M., Ohara Y., Edahiro A., Kawai H., Fujiwara Y., Kim H., Ihara K. (2022). Oral hypofunction and malnutrition among community-dwelling older adults: Evidence from the Otassha study. Gerodontology.

[37] Tramontano A., Veronese N., Giantin V., Manzato E., Rodriguez-Hurtado D., Trevisan C., De Zaiacomo F., Sergi G. (2016). Nutritional status, physical performance and disability in the elderly of the Peruvian Andes. Aging Clin. Exp. Res..

[38] Ramsey K.A., Meskers C.G.M., Trappenburg M.C., Verlaan S., Reijnierse E.M., Whittaker A.C., Maier A.B. (2020). Malnutrition is associated with dynamic physical performance. Aging Clin. Exp. Res..

[39] Nagai K., Komine T., Ikuta M., Gansa M., Matsuzawa R., Tamaki K., Kusunoki H., Wada Y., Tsuji S., Sano K. (2023). Decline of instrumental activities of daily living is a risk factor for nutritional deterioration in older adults: A prospective cohort study. BMC Geriatr..

[40] Wei K., Nyunt M.S.Z., Gao Q., Wee S.L., Ng T.P. (2019). Long-term changes in nutritional status are associated with functional and mortality outcomes among community-living older adults. Nutrition.

